# Supplementary figures and images for: Sol-Gel-Derived Fibers Based on Amorphous α-Hydroxy-Carboxylate-Modified Titanium(IV) Oxide as a 3-Dimensional Scaffold
Source: Materials (Basel). 2022 Apr 8;15(8):2752. doi: 10.3390/ma15082752 (PMC9024846; doi:10.3390/ma15082752)

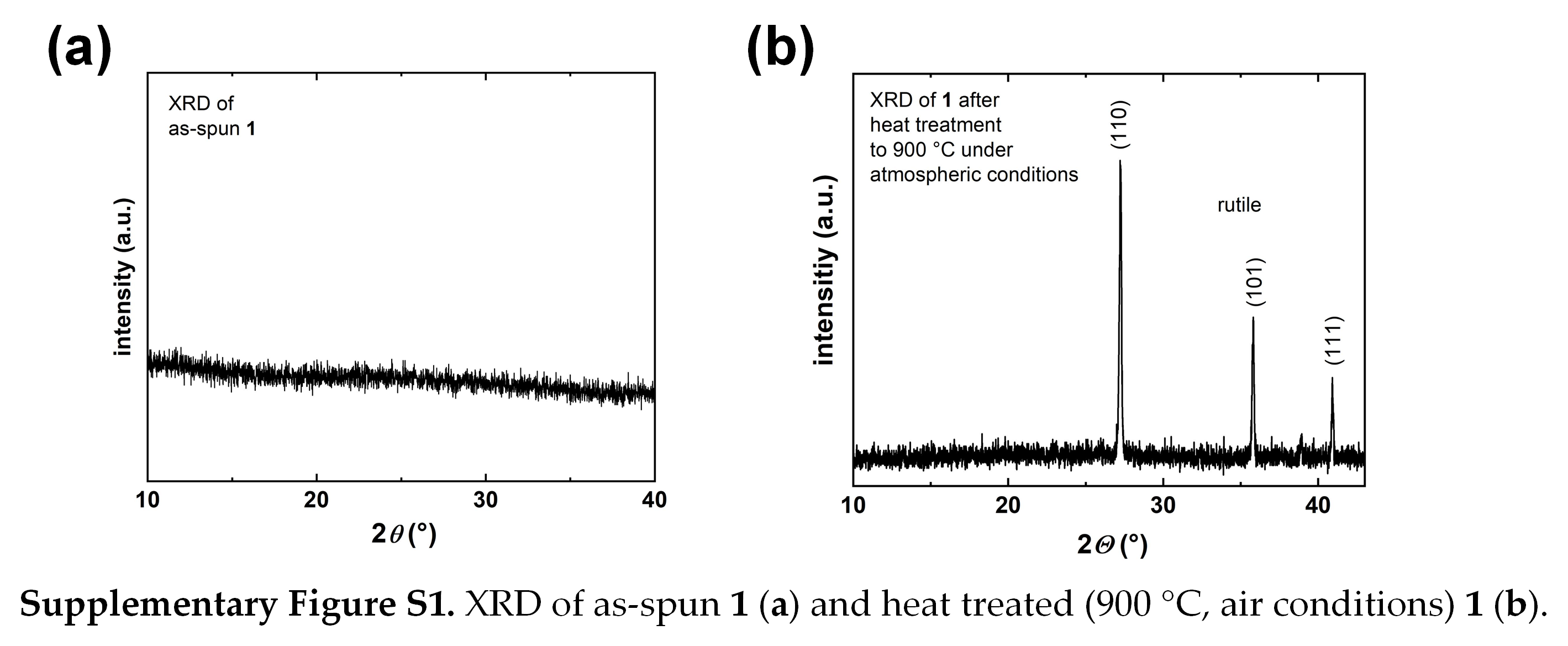

Supplement: Supplementary file 1 [file materials-15-02752-s001.zip › Supplementary Figure S1.tif]

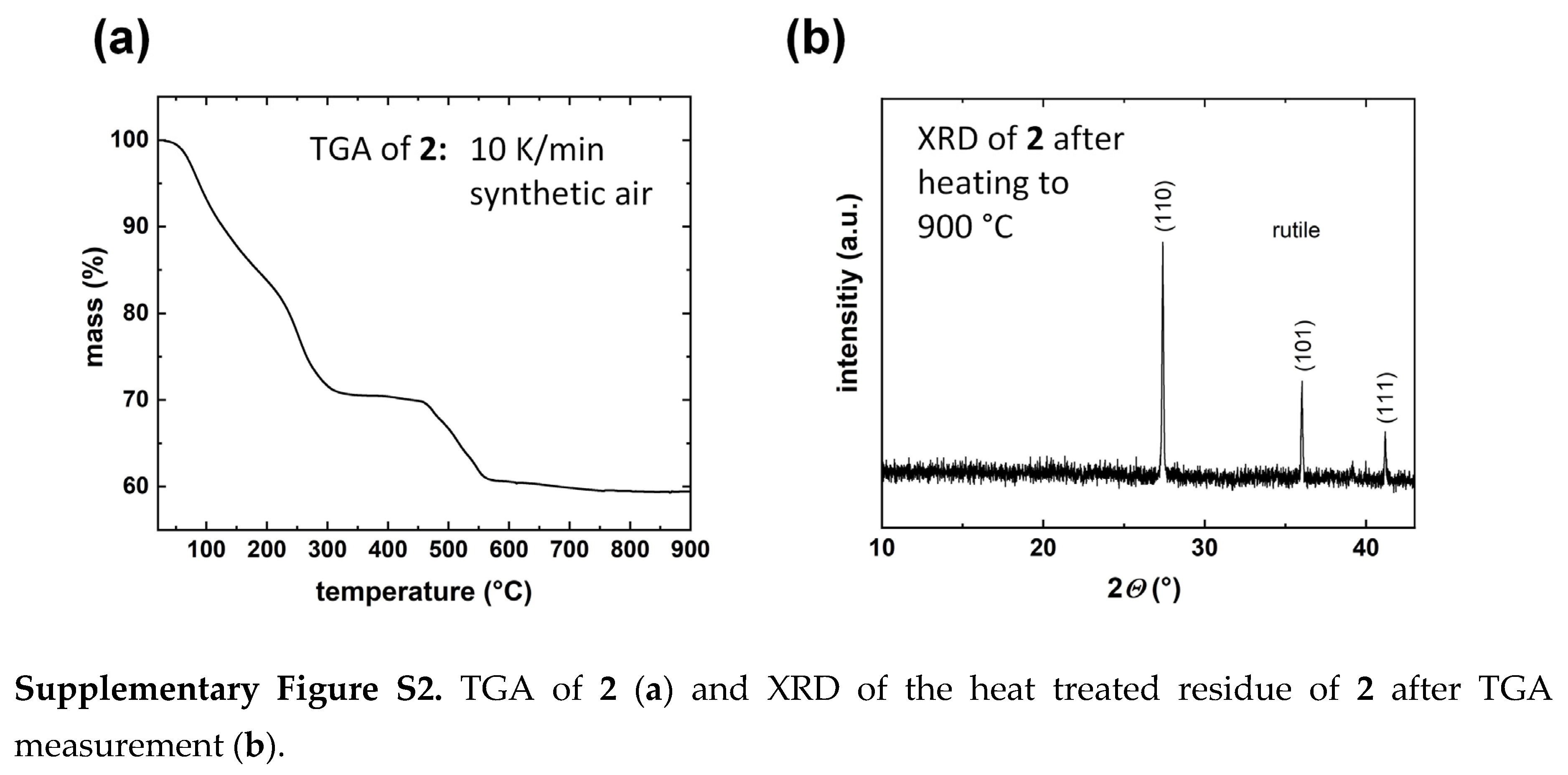

Supplement: Supplementary file 1 [file materials-15-02752-s001.zip › Supplementary Figure S2.tif]

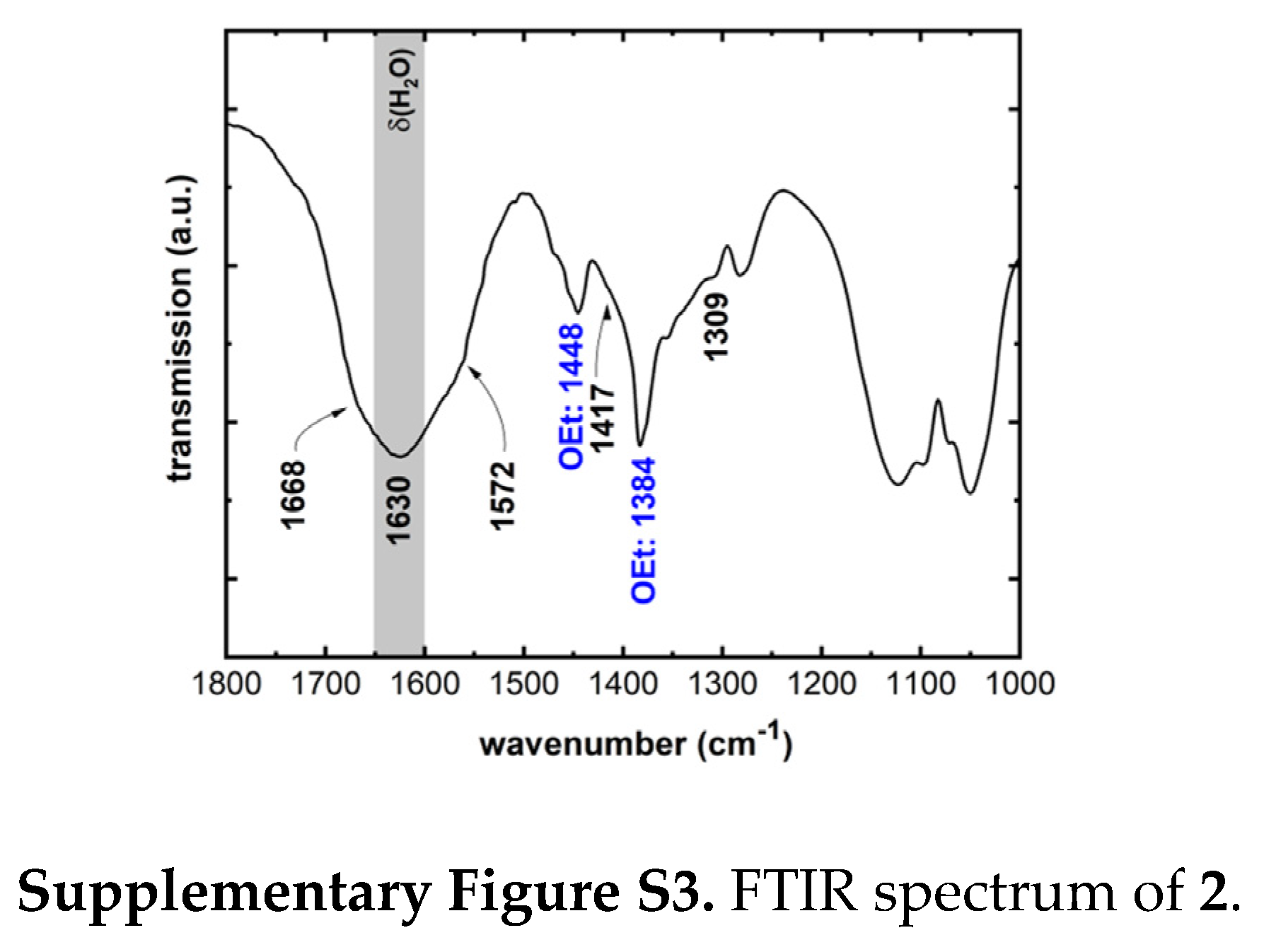

Supplement: Supplementary file 1 [file materials-15-02752-s001.zip › Supplementary Figure S3.tif]
